# 2-[(*E*)-(3,4-Dimethyl­isoxazol-5-yl)imino­meth­yl]phenol

**DOI:** 10.1107/S1600536810008160

**Published:** 2010-03-06

**Authors:** Hoong-Kun Fun, Madhukar Hemamalini, Abdullah M. Asiri, Salman A. Khan, Khalid A. Khan

**Affiliations:** a X-ray Crystallography Unit, School of Physics, Universiti Sains Malaysia, 11800 USM, Penang, Malaysia; b Department of Chemistry, Faculty of Science, King Abdu Aziz University, Jeddah, Saudi Arabia

## Abstract

The title compound, C_12_H_12_N_2_O_2_, has been synthesized by the reaction of 5-amino-3,4-dimethyl­isoxazole and salicyladehyde. The mol­ecule adopts an *E* configuration about the central C=N double bond. The dihedral angle between the isoxazole and phenyl rings is 4.2 (2)° and an intra­molecular O—H⋯N hydrogen bond generates an *S*(6) ring motif. The crystal studied was a non-merohedral twin with a domain ratio of 0.834 (4):0.166 (4).

## Related literature

For background to the biological and pharmacological properties of oxazole derivatives, see: Spinelli (1999 ▶); Conti *et al.* (1998 ▶); Mishra *et al.* (1998 ▶); Ko *et al.* (1998 ▶); Kang *et al.* (2000 ▶); Huang & Chen (2005 ▶). For details of hydrogen bonding and hydrogen-bond motifs, see: Jeffrey & Saenger (1991 ▶); Bernstein *et al.* (1995 ▶); Jeffrey (1997 ▶); Scheiner (1997 ▶). For the stability of the temperature controller used in the data collection, see: Cosier & Glazer (1986 ▶).
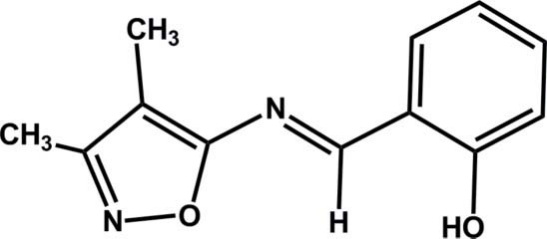


## Experimental

### 

#### Crystal data

C_12_H_12_N_2_O_2_
*M* *_r_* = 216.24
Triclinic, 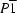

*a* = 5.3475 (14) Å
*b* = 8.615 (2) Å
*c* = 12.321 (3) Å
α = 103.696 (5)°
β = 91.486 (5)°
γ = 94.059 (5)°
*V* = 549.6 (2) Å^3^
*Z* = 2
Mo *K*α radiation
μ = 0.09 mm^−1^
*T* = 100 K
0.56 × 0.14 × 0.08 mm

#### Data collection

Bruker APEX DUO CCD area-detector diffractometer
Absorption correction: multi-scan (*SADABS*; Bruker, 2009 ▶) *T*_min_ = 0.951, *T*_max_ = 0.993
2467 measured reflections
2467 independent reflections
1946 reflections with *I* > 2σ(*I*)

#### Refinement

*R*[*F* ^2^ > 2σ(*F* ^2^)] = 0.070
*wR*(*F* ^2^) = 0.203
*S* = 1.06
2467 reflections
152 parameters
H atoms treated by a mixture of independent and constrained refinement
Δρ_max_ = 0.40 e Å^−3^
Δρ_min_ = −0.34 e Å^−3^

### 

Data collection: *APEX2* (Bruker, 2009 ▶); cell refinement: *SAINT* (Bruker, 2009 ▶); data reduction: *SAINT*; program(s) used to solve structure: *SHELXTL* (Sheldrick, 2008 ▶); program(s) used to refine structure: *SHELXTL*; molecular graphics: *SHELXTL*; software used to prepare material for publication: *SHELXTL* and *PLATON* (Spek, 2009 ▶).

## Supplementary Material

Crystal structure: contains datablocks global, I. DOI: 10.1107/S1600536810008160/sj2738sup1.cif

Structure factors: contains datablocks I. DOI: 10.1107/S1600536810008160/sj2738Isup2.hkl

Additional supplementary materials:  crystallographic information; 3D view; checkCIF report

## Figures and Tables

**Table 1:** Hydrogen-bond geometry (Å, °)

*D*—H⋯*A*	*D*—H	H⋯*A*	*D*⋯*A*	*D*—H⋯*A*
O2—H1*O*2⋯N1	1.00 (9)	1.71 (8)	2.648 (5)	154 (8)

**Fig. 1.**
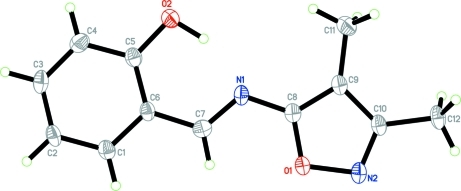
The asymmetric unit of the title compound, showing 50% probability displacement ellipsoids and the atom-numbering scheme.

**Fig. 2.**
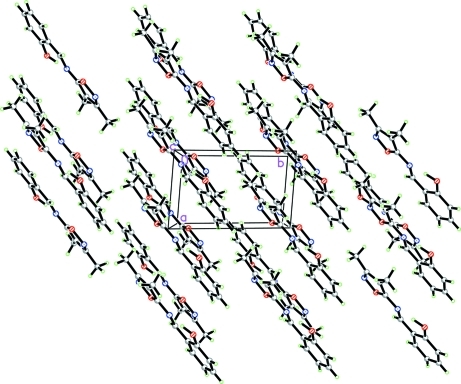
The crystal packing of the title compound (I).

## supplementary crystallographic information

### Comment

Heterocyclic compounds, especially isoxazoles, are one of the key building elements of natural products. Among the numerous heterocyclic systems of biological and pharmacological interest, the oxazole ring is endowed with various activities, including hypoglycemic (Spinelli, 1999), analgesic (Conti *et al.*, 1998), anti-inflammatory (Mishra *et al.*, 1998), anti-bacterial (Ko *et al.*, 1998) and anti-tumor (Kang *et al.*, 2000) properties.  In view of the importance of the title compound as a pharmaceutical intermediate, the paper reports its synthesis and crystal structure.

In the title compound (Fig. 1), the isoxazole ring is essentially planar with a maximum deviation of 0.002 (2) Å for atom C8. The dihedral angle between the isoxazole ring (N2/O1/C8–C10) and the phenyl ring (C1–C6) is 4.30 (15)°. The methyl groups at C9 and C10 deviate from the isoxazole mean plane by 0.056 (3) Å and 0.013 (4) Å , respectively. The C5—O2 and C7═N1 bond lengths are 1.353 (4) Å and 1.293 (4) Å, respectively, and agree with the corresponding values in 4-{[(1E)-(3,5-dibromo-2-hydroxyphenyl) methylene]-amino}-1,5-dimethyl-2-phenyl-1,2-dihydro- 3H-pyrazol-3-one [1.344 (3) and 1.292 (4) Å; Huang & Chen, 2005].

In the crystal structure (Fig. 2), the imino N atoms are linked to the phenol O atoms and act as hydrogen-bond acceptors in intramolecular O2—H1O2···N1 interactions (Table 1) (Jeffrey & Saenger, 1991; Jeffrey, 1997; Scheiner, 1997), which generate S(6) ring motifs (Bernstein *et al.*, 1995).

### Experimental

A mixture of 5-amino-3,4-dimethylisoxazole (0.50g, 0.0044 mol) and salicyladehyde (0.54g, 0.0044 mol) in methanol (15 mL) was refluxed for 5 h with stirring to give a light yellow precipitate. It was then filtered and washed with methanol to gives the pure Schiff base. Yield: 68%; mp. 116°C. IR (KBr) v_max_ cm^-1^: 2922(C—H), 1594 (C═O), 1562 (C═C), 1152 (C—N). ^1^H NMR (CDCl_3_) d: 8.89 (s, 1H, CH olefinic), 7.42 (d, H3, J=1.8Hz), 7.44 (dd, H4, J=7.8Hz), 7.02 (dd, H5, J=7.8Hz), 6.97 (d, H6, J=1.2 Hz), 2.25 (s, CH3), 2.05 (s, CH3).

### Refinement

Atom H1O2 was located from the difference Fourier map and refined freely. The remaining hydrogen atoms were positioned geometrically [C–H = 0.93 Å or 0.96 Å] and were refined using a riding model, with *U*_iso_(H) = 1.2 or 1.5 *U*_eq_(C). A rotating group model was applied to the methyl groups. The crystal is a non-merohedral twin with BASF = 0.166 (4).

### Crystal data

**Table:**

C_12_H_12_N_2_O_2_	*Z* = 2
*M**_r_* = 216.24	*F*(000) = 228
Triclinic, *P*1	*D*_x_ = 1.307 Mg m^−^^3^
Hall symbol: -P 1	Mo *K*α radiation, λ = 0.71073 Å
*a* = 5.3475 (14) Å	Cell parameters from 2862 reflections
*b* = 8.615 (2) Å	θ = 2.4–29.7°
*c* = 12.321 (3) Å	µ = 0.09 mm^−^^1^
α = 103.696 (5)°	*T* = 100 K
β = 91.486 (5)°	Plate, yellow
γ = 94.059 (5)°	0.56 × 0.14 × 0.08 mm
*V* = 549.6 (2) Å^3^	

### Data collection

**Table:**

Bruker APEX DUO CCD area-detector diffractometer	2467 independent reflections
Radiation source: fine-focus sealed tube	1946 reflections with *I* > 2σ(*I*)
graphite	*R*_int_ = 0.0000
φ and ω scans	θ_max_ = 27.5°, θ_min_ = 1.7°
Absorption correction: multi-scan (*SADABS*; Bruker, 2009)	*h* = −6→6
*T*_min_ = 0.951, *T*_max_ = 0.993	*k* = −11→10
2467 measured reflections	*l* = −6→15

### Refinement

**Table:**

Refinement on *F*^2^	Primary atom site location: structure-invariant direct methods
Least-squares matrix: full	Secondary atom site location: difference Fourier map
*R*[*F*^2^ > 2σ(*F*^2^)] = 0.070	Hydrogen site location: inferred from neighbouring sites
*wR*(*F*^2^) = 0.203	H atoms treated by a mixture of independent and constrained refinement
*S* = 1.06	*w* = 1/[σ^2^(*F*_o_^2^) + (0.0657*P*)^2^ + 1.1217*P*] where *P* = (*F*_o_^2^ + 2*F*_c_^2^)/3
2467 reflections	(Δ/σ)_max_ = 0.001
152 parameters	Δρ_max_ = 0.40 e Å^−^^3^
0 restraints	Δρ_min_ = −0.34 e Å^−^^3^

### Special details

**Table:**

Experimental. The crystal was placed in the cold stream of an Oxford Cryosystems Cobra open-flow nitrogen cryostat (Cosier & Glazer, 1986) operating at 100.0 (1) K.
Geometry. All s.u.'s (except the s.u. in the dihedral angle between two l.s. planes) are estimated using the full covariance matrix. The cell s.u.'s are taken into account individually in the estimation of s.u.'s in distances, angles and torsion angles; correlations between s.u.'s in cell parameters are only used when they are defined by crystal symmetry. An approximate (isotropic) treatment of cell s.u.'s is used for estimating s.u.'s involving l.s. planes.
Refinement. Refinement of F^2^ against ALL reflections. The weighted R-factor wR and goodness of fit S are based on F^2^, conventional R-factors R are based on F, with F set to zero for negative F^2^. The threshold expression of F^2^ > 2σ(F^2^) is used only for calculating R-factors(gt) etc. and is not relevant to the choice of reflections for refinement. R-factors based on F^2^ are statistically about twice as large as those based on F, and R- factors based on ALL data will be even larger.

### Fractional atomic coordinates and isotropic or equivalent isotropic displacement parameters (Å^2^)

**Table:**

	*x*	*y*	*z*	*U*_iso_*/*U*_eq_	
O1	1.0387 (4)	1.1305 (2)	0.41739 (17)	0.0228 (5)	
O2	0.6135 (4)	0.7350 (3)	0.08828 (18)	0.0262 (5)	
N1	0.8330 (5)	0.9521 (3)	0.2591 (2)	0.0194 (5)	
N2	1.2481 (5)	1.2478 (3)	0.4414 (2)	0.0252 (6)	
C1	0.2930 (5)	0.7387 (3)	0.3499 (3)	0.0201 (6)	
H1A	0.3082	0.7892	0.4256	0.024*	
C2	0.0960 (5)	0.6240 (3)	0.3108 (3)	0.0221 (6)	
H2A	−0.0224	0.5990	0.3593	0.026*	
C3	0.0782 (6)	0.5468 (4)	0.1976 (3)	0.0249 (7)	
H3A	−0.0529	0.4691	0.1710	0.030*	
C4	0.2512 (6)	0.5831 (4)	0.1235 (3)	0.0250 (7)	
H4A	0.2362	0.5296	0.0484	0.030*	
C5	0.4491 (5)	0.7008 (3)	0.1626 (2)	0.0199 (6)	
C6	0.4711 (5)	0.7802 (3)	0.2771 (2)	0.0186 (6)	
C7	0.6723 (5)	0.9024 (3)	0.3225 (2)	0.0191 (6)	
H7A	0.6859	0.9462	0.3992	0.023*	
C8	1.0225 (5)	1.0682 (3)	0.3052 (2)	0.0176 (6)	
C9	1.2091 (5)	1.1366 (3)	0.2539 (2)	0.0192 (6)	
C10	1.3428 (5)	1.2479 (3)	0.3446 (2)	0.0195 (6)	
C11	1.2629 (6)	1.1010 (4)	0.1327 (3)	0.0276 (7)	
H11A	1.1457	1.0163	0.0921	0.041*	
H11C	1.4305	1.0685	0.1229	0.041*	
H11D	1.2470	1.1953	0.1051	0.041*	
C12	1.5687 (5)	1.3582 (4)	0.3386 (3)	0.0243 (7)	
H12A	1.6191	1.4202	0.4124	0.036*	
H12D	1.5285	1.4287	0.2920	0.036*	
H12B	1.7034	1.2963	0.3075	0.036*	
H1O2	0.722 (11)	0.820 (7)	0.132 (5)	0.080 (18)*	

### Atomic displacement parameters (Å^2^)

**Table:**

	*U*^11^	*U*^22^	*U*^33^	*U*^12^	*U*^13^	*U*^23^
O1	0.0194 (11)	0.0238 (11)	0.0233 (11)	−0.0060 (8)	0.0019 (8)	0.0042 (8)
O2	0.0250 (12)	0.0281 (11)	0.0232 (11)	−0.0040 (9)	0.0061 (9)	0.0028 (9)
N1	0.0170 (12)	0.0148 (11)	0.0261 (13)	0.0009 (9)	0.0020 (9)	0.0041 (9)
N2	0.0207 (13)	0.0246 (13)	0.0290 (14)	−0.0061 (10)	−0.0003 (10)	0.0062 (10)
C1	0.0155 (14)	0.0176 (13)	0.0270 (15)	0.0054 (10)	0.0036 (11)	0.0036 (11)
C2	0.0152 (14)	0.0204 (14)	0.0318 (16)	0.0021 (11)	0.0054 (11)	0.0078 (12)
C3	0.0159 (14)	0.0225 (14)	0.0353 (17)	−0.0013 (11)	−0.0012 (12)	0.0063 (12)
C4	0.0247 (16)	0.0227 (15)	0.0246 (15)	−0.0006 (12)	−0.0007 (12)	0.0004 (12)
C5	0.0164 (14)	0.0194 (14)	0.0242 (14)	0.0026 (11)	0.0021 (11)	0.0052 (11)
C6	0.0154 (13)	0.0155 (13)	0.0256 (14)	0.0033 (10)	0.0019 (11)	0.0054 (11)
C7	0.0199 (14)	0.0158 (13)	0.0216 (14)	0.0058 (11)	0.0019 (11)	0.0034 (10)
C8	0.0140 (13)	0.0163 (13)	0.0237 (14)	0.0051 (10)	0.0029 (10)	0.0056 (10)
C9	0.0159 (13)	0.0169 (13)	0.0262 (15)	0.0037 (10)	0.0033 (11)	0.0074 (11)
C10	0.0136 (13)	0.0169 (13)	0.0296 (15)	0.0038 (10)	0.0020 (11)	0.0079 (11)
C11	0.0272 (16)	0.0291 (16)	0.0260 (16)	0.0009 (13)	0.0080 (12)	0.0055 (12)
C12	0.0137 (13)	0.0215 (14)	0.0392 (17)	0.0005 (11)	0.0038 (12)	0.0100 (12)

### Geometric parameters (Å, °)

**Table:**

O1—C8	1.357 (3)	C4—H4A	0.9300
O1—N2	1.429 (3)	C5—C6	1.412 (4)
O2—C5	1.353 (4)	C6—C7	1.452 (4)
O2—H1O2	0.95 (6)	C7—H7A	0.9300
N1—C7	1.293 (4)	C8—C9	1.367 (4)
N1—C8	1.381 (4)	C9—C10	1.426 (4)
N2—C10	1.308 (4)	C9—C11	1.491 (4)
C1—C2	1.385 (4)	C10—C12	1.498 (4)
C1—C6	1.410 (4)	C11—H11A	0.9600
C1—H1A	0.9300	C11—H11C	0.9600
C2—C3	1.394 (4)	C11—H11D	0.9600
C2—H2A	0.9300	C12—H12A	0.9600
C3—C4	1.387 (4)	C12—H12D	0.9600
C3—H3A	0.9300	C12—H12B	0.9600
C4—C5	1.404 (4)		
			
C8—O1—N2	107.7 (2)	N1—C7—H7A	119.1
C5—O2—H1O2	103 (3)	C6—C7—H7A	119.1
C7—N1—C8	120.3 (2)	O1—C8—C9	110.8 (2)
C10—N2—O1	105.3 (2)	O1—C8—N1	119.7 (2)
C2—C1—C6	121.2 (3)	C9—C8—N1	129.5 (3)
C2—C1—H1A	119.4	C8—C9—C10	103.2 (3)
C6—C1—H1A	119.4	C8—C9—C11	128.4 (3)
C1—C2—C3	118.9 (3)	C10—C9—C11	128.4 (3)
C1—C2—H2A	120.6	N2—C10—C9	112.9 (3)
C3—C2—H2A	120.6	N2—C10—C12	119.8 (3)
C4—C3—C2	121.6 (3)	C9—C10—C12	127.3 (3)
C4—C3—H3A	119.2	C9—C11—H11A	109.5
C2—C3—H3A	119.2	C9—C11—H11C	109.5
C3—C4—C5	119.7 (3)	H11A—C11—H11C	109.5
C3—C4—H4A	120.2	C9—C11—H11D	109.5
C5—C4—H4A	120.2	H11A—C11—H11D	109.5
O2—C5—C4	118.4 (3)	H11C—C11—H11D	109.5
O2—C5—C6	121.9 (3)	C10—C12—H12A	109.5
C4—C5—C6	119.6 (3)	C10—C12—H12D	109.5
C1—C6—C5	119.0 (3)	H12A—C12—H12D	109.5
C1—C6—C7	118.8 (3)	C10—C12—H12B	109.5
C5—C6—C7	122.2 (3)	H12A—C12—H12B	109.5
N1—C7—C6	121.8 (3)	H12D—C12—H12B	109.5
			
C8—O1—N2—C10	−0.3 (3)	N2—O1—C8—C9	0.3 (3)
C6—C1—C2—C3	1.3 (4)	N2—O1—C8—N1	−179.4 (2)
C1—C2—C3—C4	−0.6 (5)	C7—N1—C8—O1	−0.3 (4)
C2—C3—C4—C5	−0.3 (5)	C7—N1—C8—C9	−179.9 (3)
C3—C4—C5—O2	−179.3 (3)	O1—C8—C9—C10	−0.2 (3)
C3—C4—C5—C6	0.5 (4)	N1—C8—C9—C10	179.4 (3)
C2—C1—C6—C5	−1.1 (4)	O1—C8—C9—C11	178.7 (3)
C2—C1—C6—C7	179.4 (3)	N1—C8—C9—C11	−1.7 (5)
O2—C5—C6—C1	180.0 (3)	O1—N2—C10—C9	0.1 (3)
C4—C5—C6—C1	0.2 (4)	O1—N2—C10—C12	−179.8 (2)
O2—C5—C6—C7	−0.5 (4)	C8—C9—C10—N2	0.0 (3)
C4—C5—C6—C7	179.7 (3)	C11—C9—C10—N2	−178.9 (3)
C8—N1—C7—C6	−179.7 (2)	C8—C9—C10—C12	180.0 (3)
C1—C6—C7—N1	−176.1 (3)	C11—C9—C10—C12	1.1 (5)
C5—C6—C7—N1	4.4 (4)		

### Hydrogen-bond geometry (Å, °)

**Table:**

*D*—H···*A*	*D*—H	H···*A*	*D*···*A*	*D*—H···*A*
O2—H1O2···N1	1.00 (9)	1.71 (8)	2.648 (5)	154 (8)
